# 

**Published:** 1885-03

**Authors:** 


### National Association of Dental Examiners.

The third meeting of the National Association of Dental Examiners will be held at New Orleans, on Tuesday, March 31st, 1885.

All State Boards not already members of this Association, are earnestly invited to send representatives to this meeting.

GEO. H. CUSHING, Secretary.

### Southern Dental Association.

The seventeenth annual session of the Southern Dental Association will be held in New Orleans, commencing Tuesday, March 31st, 1885, and continue four days.

This promises to be the largest and best meeting ever held in the South.

Railroad travel to New Orleans is now unprecedentedly low, and the attractions to the World's Exposition are certainly very great—sufficient to induce almost every one to make a visit to the Crescent City. The display of dental instruments will surpass any heretofore made.

The Committee of Arrangements will see that ample provision is made for the accommodation of all who may come. Those wishing to engage rooms in advance will correspond with Dr. J. R. Walker, Chairman of the Committee of Arrangements, corner of Barone and Commerce sts., New Orleans.

The following subjects will be presented for consideration, viz.:

1. Dental Education.
2. Hygiene.
3. Pathology and Therapeutics.
4. Histology and Microscopy.
5. Chemistry.
6. Operative Dentistry.
7. Medical Dentistry.
8. Dental Literature.
9. Appliances and Improvements.

Ample provision wili be made for clinics, and those attending may expect a rare treat, as some of the most prominent men in the profession will be present for that purpose. All the novelties and latest designs in instruments will be exhibited, and their use practically demonstrated.

It is expected that members of the profession from all parts of the country will be present, and make the occasion one of pleasure and profit. No more pleasant and inviting time of the year could be selected for making a visit to the South than the first of April.

### Ohio State Dental Society.—Reorganized.

Below is given a list of the officers and committees of the Ohio State Dental Society, as reorganized at the time of the last meeting in October, 1884. It is very desirable that the various committees should confer, and each prepare its work for the next annual meeting, to be held next October, in Chillicothe, when it is earnestly hoped there will be a good meeting.

President, C. H. James, Cincinnati; Vice President, H. H. Harrison, Cadiz; Secretary, J. R. Callahan, Hillsboro; Treasurer, Geo. W. Keely, Oxford.

STATE BOARD OF EXAMINERS.

H. A. Smith, 1887; C. R. Butler, 1886; I. Williams, 1885; J. Taft, 1886; F. H. Rehwinkel, 1885.

STANDING COMMITTEES.

Executive.—F. H. Rehwinkel, (Chm.) Chillicothe; C. R. Butler, Cleveland; E. G. Betty, Cincinnati.

VOLUNTARY ESSAYS.

J. Taft, (Chm.) Cincinnati; George Watt, Xenia; D. Gibbons, Warren.

PUBLICATION.

W. H. Sillito, (Chm.) Xenia; J. R. Callahan, Hillsboro; H. H. Harrison, Cadiz.

ETHICS.

F. H. Rehwinkel, (Chm.) Chillicothe; C. H. James, Cincinnati; I. Williams, New Philadelphia.

MEMBERSHIP.

C. H. Harroun, (Chm,) Toledo; A. F. Eminger, Columbus; G. W. Keely, Oxford.

REVISION OF STATE LAW.

F. H. Rehwinkel, (Chm.) Chillicothe; Frank A. Hunter, Cincinnati; C. H. James, Cincinnati.

### Meeting.

The thirtieth annual meeting of the Michigan State Dental Society, will be held in the city of Detroit, beginning March 25th, at 7½ o'clock P. M., and continue two days. The programme for the meeting contains subjects of great interest to all members of the dental profession, and it is desirable that members, each one, as far as possible, prepare a paper on one or more of the subjects. Indeed, let all come prepared to take some active part in the work of the meeting. Those who are not members of the Society are cordially invited to attend. The following are the subjects for consideration:

1. Filling Teeth. Materials, Methods, and Instruments.
2. Our Society; how can its usefulness be increased.
3. Treatment of Diseased Teeth, Gums, and Pulps.
4. Dental Education.
5. Prosthetic Dentistry.
6. Office Practice.

The State Board of Dental Examiners will meet in Detroit at the same time.

### THE DENTAL REGISTER,

A MONTHLY MAGAZINE DEVOTED TO THE INTERESTS OF THE

### DENTAL PROFESSION.

*Terms Reduced to* $2.00 *Per Year. Single Copies,* 25 *Cents.*

**EDITED BY PROF. J. TAFT, D.D.S.**

—AND—

**Published by SPENCER & CROCKER, Ohio Dental and Surgical Depot.**

The volume commences in January and closes in December.

The REGISTER being issued monthly is always fresh, therefore Indispensable to the practitioner who desires to keep posted up with the new inventions and improvements which are daily developed. Neither expense nor effort will be spared to give value and variety to its contents. Articles will be illustrated with well executed engravings whenever they will add to the interest or assist to explanation.

Its extensive circulation and frequency of issue make it one of the BEST DENTAL ADVERTISING MEDIUMS in the United States.

All subscriptions must begin with January or July.

Local or special notices, five lines or less, will be inserted for $3 each insertion; over five lines, 50 cts. per line.

All advertising bills payable in advance, or at furthest, after the issue of the first number containing the advertisement. All transient advertisements to be paid for in advance.

☞CONTRIBUTIONS SOLICITED.

Exclusively Editorial Communications should be addressed to the Editor.

The latest number of the REGISTER may be ordered with or without other goods at any time, and all letters should be addressed to

**SPENCER & CROCKER, Ohio Dental & Surgical Depot, Cincinnati, O.**

